# Organization of the *Escherichia coli* aerobic enzyme complexes of oxidative phosphorylation in dynamic domains within the cytoplasmic membrane

**DOI:** 10.1002/mbo3.163

**Published:** 2014-04-12

**Authors:** Heiko Erhardt, Felix Dempwolff, Moritz Pfreundschuh, Marc Riehle, Caspar Schäfer, Thomas Pohl, Peter Graumann, Thorsten Friedrich

**Affiliations:** 1 Institut für Biochemie, Albert-Ludwigs-UniversitätAlbertstraße 21, Freiburg, 79104, Germany; 2 Mikrobiologie, Institut für Biologie II, Albert-Ludwigs-UniversitätSchänzlestraße 1, Freiburg, 79104, Germany; 3 LOEWE Center for Synthetic Microbiology (SYNMICRO), Philipps-UniversitätHans-Meerwein-Straße 6, Marburg, 35043, Germany; 4 Department of Biosystems Science and Engineering, ETH ZürichMattenstraße 26, Basel, 4058, Switzerland; 5 Institut für Pharmakologie und Toxikologie, Eberhard-Karls-UniversitätWilhelmstraße 56, Tübingen, 72074, Germany; 6 Institut für Biologie und Mikrobiologie, Humboldt Universität zu BerlinChausseestraße 117, Berlin, 10115, Germany; 7 SuppreMol GmbHAm Klopferspitz 19, Martinsried, 82152, Germany

**Keywords:** *Escherichia coli*, FRAP, in vivo fluorescence microsocopy, membrane protein organization, oxidative phosphorylation, TIRF microscopy

## Abstract

The *Escherichia coli* cytoplasmic membrane contains the enzyme complexes of oxidative phosphorylation (OXPHOS). Not much is known about their supramolecular organization and their dynamics within the membrane in this model organism. In mitochondria and other bacteria, it was demonstrated by nondenaturing electrophoretic methods and electron microscopy that the OXPHOS complexes are organized in so-called supercomplexes, stable assemblies with a defined number of the individual enzyme complexes. To investigate the organization of the *E. coli* enzyme complexes of aerobic OXPHOS in vivo, we established fluorescent protein fusions of the NADH:ubiquinone oxidoreductase, the succinate:ubiquinone oxidoreductase, the cytochrome *bd*-I, and the cytochrome *bo*_*3*_ terminal oxidases, and the F_o_F_1_ ATP-synthase. The fusions were integrated in the chromosome to prevent artifacts caused by protein overproduction. Biochemical analysis revealed that all modified complexes were fully assembled, active, and stable. The distribution of the OXPHOS complexes in living cells was determined using total internal reflection fluorescence microscopy. The dynamics within the membrane were detected by fluorescence recovery after photobleaching. All aerobic OXPHOS complexes showed an uneven distribution in large mobile patches within the *E. coli* cytoplasmic membrane. It is discussed whether the individual OXPHOS complexes are organized as clustered individual complexes, here called “segrazones.”

## Introduction

The aerobic oxidative phosphorylation (OXPHOS) in *Escherichia coli* is mainly catalyzed by six enzyme complexes located in the cytoplasmic membrane. Five oxidoreductases transfer electrons from NADH and succinate to oxygen. In doing so, a proton gradient across the membrane is generated that is needed for energy-consuming processes such as ATP synthesis catalyzed by the sixth enzyme complex, the F_o_F_1_ ATP-synthase (Ingledew and Poole 1984; Senior et al. 2002; Price and Driessen 2010). The NADH:ubiquinone oxidoreductase (complex I), the alternative NADH dehydrogenase and the succinate:ubiquinone oxidoreductase (complex II) are the primary dehydrogenases acting as entry points for electrons from NADH and succinate into the respiratory chain (Friedrich 2001; Cecchini et al. 2002; Feng et al. 2012). In contrast to complex I, the reactions of the alternative NADH dehydrogenase and of complex II are not coupled with the translocation of protons across the membrane. However, the terminal cytochrome *bd*-I (Borisov et al. 2011) and cytochrome *bo* oxidases (Abramson et al. 2000) couple the reduction of oxygen to water with the generation of a proton gradient across the membrane. The primary dehydrogenases and the terminal oxidases are connected by the mobile carrier ubiquinone. Under microaerophilic and anoxic conditions menaquinone and demethylmenaquinone are used as electron carriers (Unden and Bongaerts 1997). The complexes investigated in this study are listed in Table 1.

**Table 1:** The *Escherichia coli* aerobic OXPHOS enzyme complexes investigated in this study

Enzyme complex	Mol. mass (kDa)	Number of different subunits	Genes	FP fusions in *E. coli* BW25113 (localization)
NADH:ubiquinone oxidoreductase (complex I)	535	13	*nuoA-nuoN*	*nuoF-mcerulean* *mcherry-nuoF* *egfp-nuoF* (all cytoplasm)
Succinate:ubiquinone oxidoreductase (complex II)	120	4	*sdhCDAB*	*mcherry-sdhC* (cytoplasm)
Cytochrome *bd-*I complex	100	2	*cydA-cydB*	*cydB-egfp* (cytoplasm)
Cytochrome *bo*_*3*_ complex	140	2	*cyoA-cyoD*	*cyoA-mcherry* (periplasm)
F_o_F_1_ ATP-synthase	528	8	*atpA-atpH*	*atpB-egfp* (cytoplasm)

An early description of the organization of membrane proteins within the biological membrane is the “fluidic mosaic model” depicting the membrane as a two-dimensional phase in which the membrane proteins and hydrophobic electron carriers freely diffuse (Singer and Nicolson 1972). The concept of proteins freely diffusing in the membrane was challenged by fluorescence microscopy experiments (Jacobson et al. 1995; Mika and Poolman 2011) and the detection of membrane lipid domains (Groves 2006; Matsumoto et al. 2006). Furthermore, the model was questioned by experiments demonstrating a higher order organization of membrane proteins in so-called supercomplexes, stable assemblies containing a defined stoichiometry of individual complexes. The existence of supercomplexes was shown in mitochondria from several species (Schägger and Pfeiffer 2000; Zhang et al. 2005; Nübel et al. 2009; Lenaz et al. 2010) and in bacteria such as *Paracoccus denitrificans* (Stroh et al. 2004) by nondenaturing PAGE (polyacrylamide gel electrophoresis) techniques, by electron microscopy, (Dudkina et al. 2010, 2011; Davies et al. 2012) and the biochemical preparation of supercomplexes (Niebisch and Bott 2003). The formation of supercomplexes might help to enhance the stability of the individual complexes and might accelerate the reaction rates by substrate channeling (Matsumoto et al. 2006; Romantsov et al. 2010).

Here, we investigate the localization and dynamics of the individual OXPHOS complexes in the *E. coli* cytoplasmic membrane in vivo by fluorescence microscopy. To avoid artifacts possibly caused by overproduction, chromosomally encoded fusions of the OXPHOS complexes with various fluorescent proteins (FP) were established by *λ*-red-mediated recombination (Datsenko and Wanner 2000; Pohl et al. 2007). We integrated the genes encoding eGFP (enhanced GFP), mCerulean, and mCherry (Ito et al. 1999; Rizzo et al. 2004; Shu et al. 2006) into the *nuo*-, *sdh*-, *cyd*-, *cyo-,* and *atp*-operon in the *E. coli* chromosome. In order not to disturb the assembly of the complexes and not to influence the enzymatic activity, proper positions for the FP fusions had to be identified. In vivo fluorescence microscopy with the strains revealed that the enzyme complexes in question are unevenly distributed in the *E. coli* cytoplasmic membrane in large patches. Their distribution patterns and mobility in the membrane were similar in TIRF (Groves et al. 2008) and FRAP (Mullineaux 2004) experiments.

## Experimental Procedures

### Strains, plasmids and oligonucleotides

All *E. coli* strains, plasmids, and oligonucleotides used in this work are listed in Tables S1, S2, and S3, respectively.

### Cell growth

Cells were grown either in LB medium or in M9 minimal medium containing succinate as sole carbon source. For fluorescence microscopy 4 mL S_750_-medium was inoculated in a 1:100 ratio (v:v) with a 4 mL overnight culture in LB medium and grown for 3–5 h at 30°C. To select the desired mutations, cells were grown in the presence of 100 *μ*g mL^−1^ ampicillin, 170 *μ*g mL^−1^ (for liquid media) or 20 *μ*g mL^−1^ (for agar plates) chloramphenicol or 50 *μ*g mL^−1^ kanamycin.

### Activity assays

The NADH and succinate oxidase activity of cytoplasmic membranes was measured with a Clarke-type oxygen electrode at 30°C in a volume of 2 mL. The assay contained 2–3 mg cytoplasmic membranes and the reaction was initiated by the addition of the corresponding substrate after obtaining a constant baseline. The “substrate”/ferricyanide oxidoreductase activities were measured as the decrease in the absorbance of ferricyanide at 410 nm with a Ultraspec spectrophotometer (Amersham Pharmacia Biotech, Munich, Germany) in a volume of 1 mL. The assay contained 10 *μ*L membrane suspension or 30 *μ*L of a fraction after sucrose gradient centrifugation. Detailed conditions are provided in the Data S1.

### Sucrose gradient centrifugation

Membrane proteins were solubilized by an addition of 3% (w/v) *n*–dodecyl-*β*-D-maltopyranoside (DDM; AppliChem, Darmstadt, Germany) at 4°C. The extract was centrifuged for 20 min at 48,000*g* and 4°C. 0.9 mL of the supernatant were loaded onto 12 mL gradients of 5–30% (w/v) sucrose and centrifuged for 18 h at 160,000*g*. The gradients were fractionated into 0.7 mL portions and the “substrate”/ferricyanide oxidoreductase activities and the fluorescence emissions of the particular FP were measured at its specific wavelength. Detailed conditions are provided in the Data S1.

### Microscopy

All strains for fluorescence microscopy were grown in S7_50_ minimal media at 30°C. Fluorescence microscopy was performed using a Zeiss Observer Z1 equipped with a 1.45 NA objective (Jena, Germany) and a Photometrix Cascade CCD camera (Munich, Germany). The fluorophores were exited by exposition to a laser beam of 488 nm, 561 nm, or 445 nm wavelengths coupled in by a visitron VisiTIRF system (Puchheim, Germany). FRAP experiments were performed using a 50 mW argon laser of 405 nm wavelength. The 50 *μ*m size of the laser beam was generated by a pinhole inserted into the laser beam within the module that incorporates the optical wire into the side port. For epifluorescence images, we used a xenon mercury burner with appropriate filter sets. Images were processed with the Metamorph 7.5.5 software (Sunnyvale, CA). For the TIRF- and FRAP-streams, every picture was optimized for the best signal to noise ratio.

## Results

### Integration of the FP-gene fusions in the *E. coli* BW25113 chromosome

For in vivo localization studies, it is essential to ascertain whether the proteins in question are produced at the physiological level. Therefore, chromosomally encoded FP fusions of the aerobic OXPHOS complexes were established in *E. coli* strain BW25113 (Datsenko and Wanner 2000; Baba et al. 2006; Table 1). Individual genes comprising the FP fusion were integrated in the chromosome *via λ*-red-mediated recombination (see Supporting Information). The FP fusions were established either on subclones and introduced in the chromosome in a second step or by direct recombination of the FP sequence with the chromosome (see Supporting Information).

*Escherichia coli* complex I is made up of 13 different subunits (Friedrich 1998). It was previously shown that a His-tag fused to the N-terminus of the cytosolic subunit NuoF containing the NADH-binding site neither prevents the assembly nor the activity of complex I making NuoF a promising candidate for a FP fusion (Pohl et al. 2007). It turned out that eGFP, mCerulean, and mCherry, respectively, can be fused to both termini of NuoF. FP fusions to other subunits either prevented the assembly of the complex or led to the production of an inactive enzyme complex. Complex II consists of four subunits (Cecchini et al. 2002). The N-terminus of the membranous subunit SdhC was decorated with mCherry. FP fusions to all other subunits lead either to the formation of inclusion bodies or to the occurrence of fluorescence in the cytoplasm. The fusion of the C-terminus of the membranous subunit CydII of the cytochrome *bd*-I oxidase with an eGFP has been recently described (Lenn et al. 2008). A fluorescent variant of the cytochrome *bo*_*3*_ complex was obtained by the fusion of mCherry to the periplasmic C-terminus of CyoII. The F_o_F_1_ ATP-synthase was decorated with an eGFP at the C-terminus of subunit F_o_a encoded by *atpB* as described (Düser et al. 2008). Further experiments were performed with the *E. coli* strains BW25113 *nuoF-mcerulean*, BW25113 *mcherry-nuoF*, BW25113 *egfp-nuoF*, BW25113 *mcherry-sdhC*, BW25113 *cydB-egfp*, BW25113 *cyoA-mcherry,* and BW25113 *atpB-egfp* (Table 1). The parental strain BW25113 was used as control.

### The FP-decorated enzyme complexes are active and fully assembled

Strains containing the chromosomally encoded FP fusions showed an identical growth rate as the parental strain in LB medium and in M9-minimal medium containing succinate as sole carbon source (data not shown). The activity of the enzyme complexes decorated with the FP fusion was detected by various assays (Spehr et al. 1999). The NADH oxidase activity, a measure of the catalytic competence of complex I, and the NADH/ferricyanide oxidoreductase activity, a measure of the amount of the complex in the membrane of cytoplasmic membranes from strains BW25113 *nuoF-mcerulean*, BW25113 *mcherry-nuoF*, and BW25113 *egfp-nuoF* containing an FP fusion on complex I and from strain BW25113 was determined. The NADH oxidase activity was not decreased in the mutant with the mCherry fusion and only slightly decreased by 5% and 12% in the mutants carrying the mCerulean and the eGFP fusion, respectively (Table 2). The NADH oxidase activity was inhibited to 50% by piericidin A, a specific complex I inhibitor, in all strains in accordance with the literature (Pohl et al. 2007). The residual activity is due the presence of the alternative NADH dehydrogenase. The NADH/ferricyanide oxidoreductase activity did not vary significantly within the experimental error indicating that the amount of the complex decorated with the FP in the membrane did not change in comparison to the parental strain (Table 2).

**Table 2:** Complex I-mediated activities in the membranes of various *Escherichia coli* strains

	NADH oxidase activity	NADH/ferricyanide oxidoreductase activity
	
Strain	(*μ*mol min^−1^ mg^−1^)	
BW25513	0.43 ± 0.02	3.3 ± 0.2
BW25113 *nuoF-mcerulean*	0.41 ± 0.04	3.2 ± 0.1
BW25113 *mcherry-nuoF*	0.44 ± 0.01	3.0 ± 0.1
BW25113 *egfp-nuoF*	0.38 ± 0.02	3.4 ± 0.6

The succinate oxidase activity mediated by complex II was reduced by one-third in strain BW25113 *mcherry-sdhC* to 0.05 ± 0.01 *μ*mol min^−1^ mg^−1^ compared to that of the parental strain with 0.08 ± 0.01 *μ*mol min^−1^ mg^−1^. However, this activity was inhibited by 20 mmol/L malonate, specifically inhibiting complex II, to more than 80% in both strains. The succinate/ferricyanide oxidoreductase activity of the FP fusion complex was decreased by 20% from 0.18 ± 0.01 *μ*mol min^−1^ mg^−1^ to 0.15 ± 0.01*μ*mol min^−1^ mg^−1^ due to the FP fusion. Thus, the amount of complex II in the membranes was not significantly affected by the FP fusion which in addition had only a mild effect on the physiological activity.

The NADH oxidase activity of the membranes from strain BW25113 *cydB-egfp* was 0.41 ± 0.02*μ*mol min^−1^ mg^−1^ and thus, 95% of that of the parental strain in accordance with the literature. This strain containing a functional cytochrome *bd* oxidase has been described in detail (Lenn et al. 2008). The membranes of strain BW25113 *cyoA-mCherry* producing the cytochrome *bo*_*3*_ oxidase decorated with mCherry showed an NADH oxidase activity of 0.39 ± 0.02 *μ*mol min^−1^ mg^−1^ corresponding to 90% of the parental strain activity. This activity was inhibited by 20% upon an addition of 30 mmol/L hydroxylamine. The NADH oxidase activity of membranes from strain BW25113 was inhibited by 21%. Thus, the FP fusion led only to a slight decrease in activity and to the assembly of a functional complex. The activity of ATP-synthase in strain BW25113 *atpB-egfp* was screened by its ability to grow on succinate minimal agar plates. With succinate as the sole carbon source, only cells with a functional ATP-synthase will survive. Indeed, the parental strain and strain BW25113 *atpB-egfp* were able to grow on these plates in contrast to a strain carrying an inactivated *atpB* gene in its chromosome (Fig. S1). Thus, all OXPHOS enzyme complexes decorated with an FP were active in the membrane.

**Figure 1:**
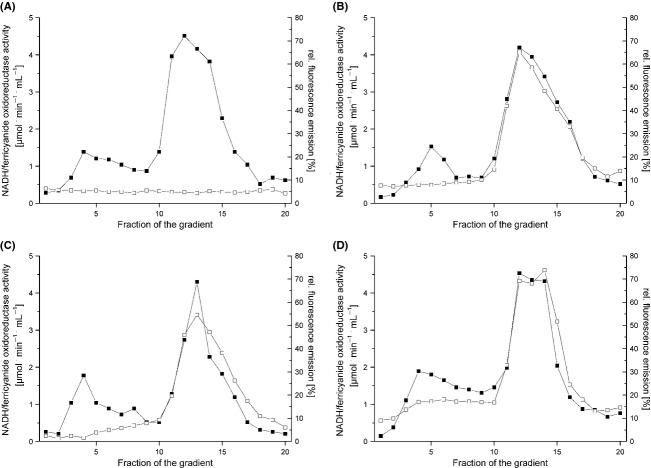
Sucrose gradient centrifugation of detergent-solubilized membranes from *E*. *coli* strains containing FP fusions on *nuoF*. The NADH/ferricyanide oxidoreductase activity (▪) and the fluorescence of the corresponding FP fusion (□) were measured in extracts from strains BW25113 (A), BW25113 *nuoF-mcerulean* (B), BW25113 *mcherry-nuoF* (C), and BW25113 *egfp-nuoF* (D). All data were normalized to 10 mg membrane protein extract applied per gradient. The fluorescence of mCerulean was measured at an excitation wavelength of 430 nm and an emission wavelength of 475 nm, that of mCherry at an excitation wavelength of 587 nm and an emission wavelength of 610 nm and that of eGFP at an excitation wavelength of 480 nm and an emission wavelength of 510 nm. The fluorescence shown in (A) was measured at an excitation wavelength of 430 nm and an emission wavelength of 475 nm. The fluorescence of the fractions obtained from strain BW25113 were measured at all three pairs of wavelength leading to similar profiles.

The assembly of the OXPHOS complexes containing the desired FP fusions was investigated by sucrose gradient centrifugation as described (Spehr et al. 1999). Membrane proteins were extracted from cytoplasmic membranes with dodecyl-maltoside and the detergent extract was loaded on a sucrose gradient (Fig. 1). After centrifugation, the gradient was fractionated and calibrated by measuring the NADH/ferricyanide and the succinate/ferricyanide oxidoreductase activity, respectively. In addition, all fractions of the gradients were characterized by the fluorescence emission of the respective FP. The NADH/ferricyanide oxidoreductase activity is caused by the fully assembled complex I with a molecular mass of 535 kDa sedimenting in fractions 11–13 under the chosen conditions. The sedimentation profiles of the NADH/ferricyanide oxidoreductase activity of extracts obtained from strains BW25113 *nuoF-mcerulean*, BW25113 *mcherry-nuoF*, and BW25113 *egfp-nuoF* were identical to the sedimentation profile of the fluorescence of the corresponding FPs (Fig. 1). This indicated that all complex I variants are fully assembled and decorated with the desired FP. The same sedimentation profile was measured with the eGFP fluorescence of the membrane extract from strain BW25113 *atpB-egfp* (Fig. 3). This is in line with the molecular mass of ATP-synthase of 528 kDa that cosediments with complex I under the chosen conditions (Spehr et al. 1999; Stolpe and Friedrich 2004).

**Figure 2:**
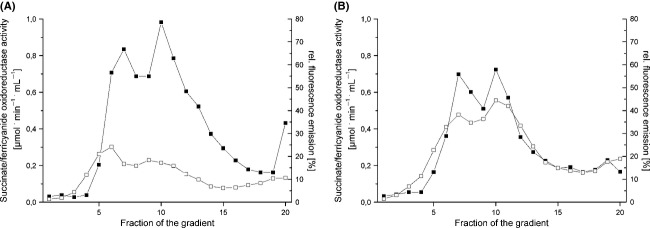
Sucrose gradient centrifugation of detergent-solubilized membranes from *E*. *coli* strains containing a FP fusion on *sdhC*. The succinate/ferricyanide oxidoreductase activity (▪) and the fluorescence of mCherry (□) were measured in extracts from strains BW25113 (A) and BW25113 *mcherry-sdhC* (B). All data were normalized to 10 mg membrane protein extract applied per gradient. The fluorescence of mCherry was measured at an excitation wavelength of 587 nm and an emission wavelength of 610 nm.

**Figure 3:**
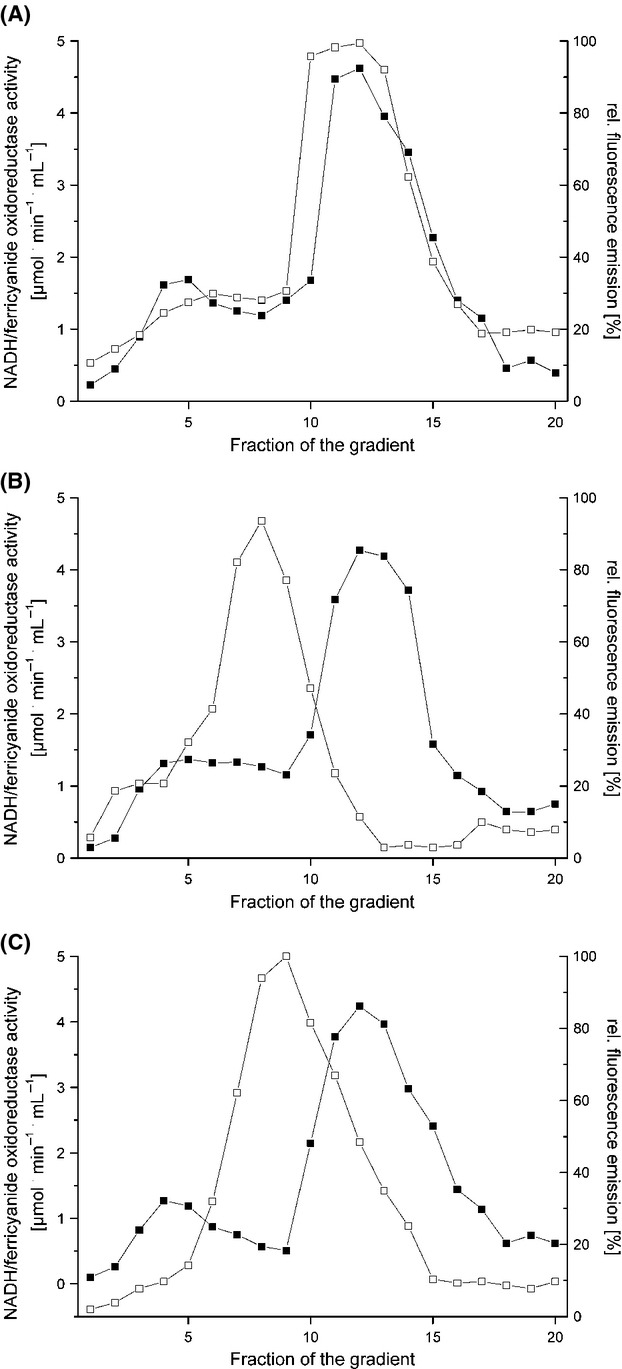
Sucrose gradient centrifugation of detergent-solubilized membranes from *E*. *coli* strains containing a FP fusion on *atpB, cydB* and *cyoA*. The NADH/ferricyanide oxidoreductase activity (▪) and the fluorescence of the corresponding FP fusion (□) were measured in extracts from strains BW25113 *atpB-egfp* (A), BW25113 *cydB-egfp* (B) and BW25113 *cyoA-mCherry* (C). All data were normalized to 10 mg membrane protein extract applied per gradient. The fluorescence of mCherry was measured at an excitation wavelength of 587 nm and an emission wavelength of 610 nm and that of eGFP at an excitation wavelength of 480 nm and an emission wavelength of 510 nm.

The succinate/ferricyanide oxidoreductase activity originates from the fully assembled monomeric complex II with a molecular mass of 120 kDa and from the homotrimer with a molecular mass of 360 kDa. The succinate/ferricyanide oxidoreductase activity of strain BW25113 *cydB-egfp* showed two activity peaks sedimenting around fraction 7 and in fractions 9–11 corresponding to the monomeric and trimeric form of complex II as described (Yankovskaya et al. 2003). The sedimentation profile of the mCherry fluorescence correlated perfectly with the activity profile exhibiting two peaks at the same positions as the activity peaks (Fig. 2). The sedimentation profile of the fluorescence emission in the extract from strains BW25113 *cydB-egfp* and BW25113 *cyoA-mCherry* showed a maximum in fractions 7–9, respectively, which is in accordance with the molecular masses of 100 and 140 kDa (Fig. 3). A fluorescence emission was not observed in any of the gradients in low molecular mass fractions corresponding to individual subunits fused with an FP excavated from a complex or to degraded proteins. Neither was fluorescence detectable in the very high molecular mass fractions corresponding to aggregates of the enzyme complexes (Figs. 1, 2 and 3). These data show that the OXPHOS complexes under investigation are fully assembled, stable, and decorated with the desired FP fusion. However, we cannot exclude the possibility that the FP fusions might have an influence on the oligomeric state of enzyme complexes.

### The OXPHOS complexes show an uneven distribution in the membrane

**Figure 4:**
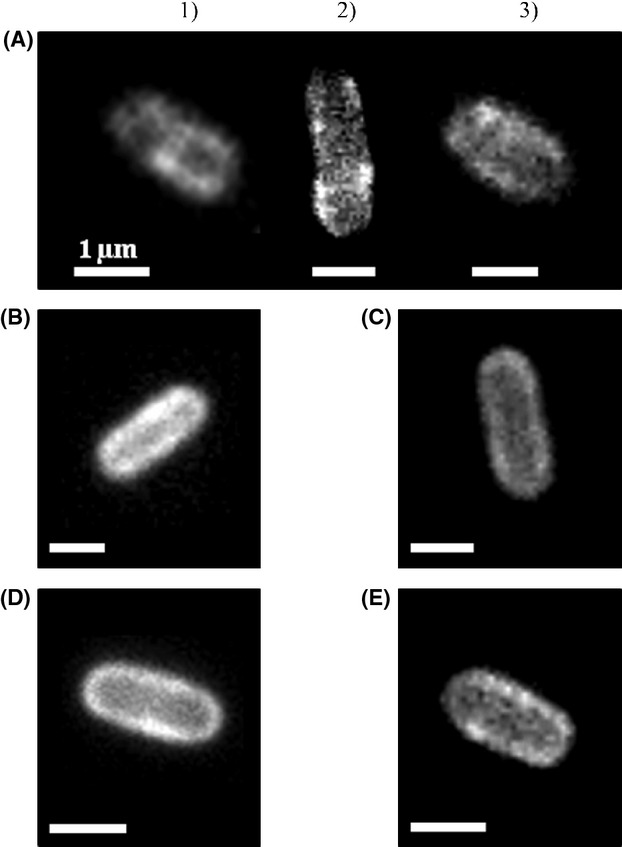
Epifluorescence images of the single cells of strains BW25113 *egfp-nuoF* (A1; 1 sec), BW25113 *nuoF-mCerulean* (A2; 1 sec), BW25113 *mcherry-nuoF* (A3; 1 sec), BW25113 *mcherry-sdhC* (B; 1 sec), BW25113 *cydB-egfp* (C; 0.6 sec), BW25113 *cyoA-mCherry* (D; 0.6 sec), and BW25113 *atpB-egfp* (E; 0.6 sec). The numbers in brackets indicate the exposure times in seconds. White bars 1 *μ*m.

The localization of the aerobic OXPHOS complexes in the membrane was determined by in vivo epifluorescence microscopy. Fluorescence emission was detected only in the cytoplasmic membrane (Fig. 4). There was no enhanced fluorescence of the cytoplasm indicating the absence of degraded or not-assembled FP fusion proteins. The FP fusions of all five protein complexes under investigation showed an uneven distribution in the membrane (Fig. 4). The fluorescence was not concentrated in specific regions of the cell, such as the cell poles as described for inclusion bodies or at the division septum as reported for members of the Min-family (Szeto et al. 2002). Compared to the other mCherry-fusions (Table 1), the fluorescence in strain BW25113 *mcherry-sdhC* was enhanced indicating a higher concentration of this complex in the *E. coli* membranes. Due to its higher concentration in the membrane, the distribution of complex II is apparently more homogeneous than in the other complexes. The patches have an estimated average diameter of 300 to 500 nm in all strains. However, due to the limited resolution of 250 nm, we cannot exclude the possibility that the patches are made up of smaller, closely spaced droplets.

### Patches of OXPHOS complexes are dynamic over time

**Figure 5:**
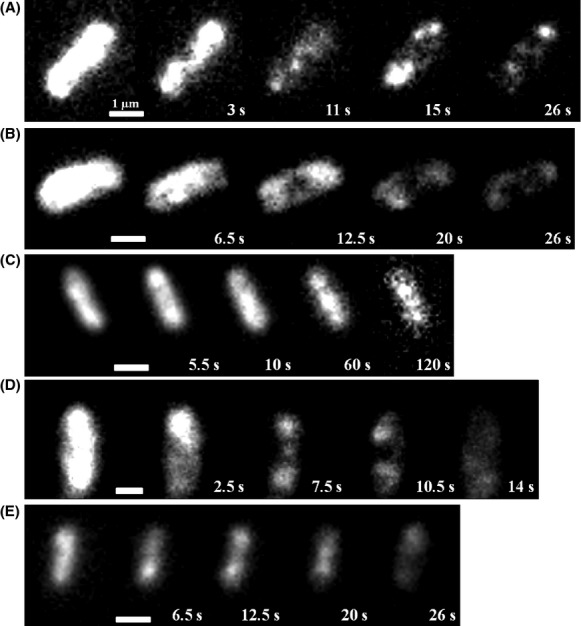
TIRF microscopy images of *E*. *coli* strains BW25113 *egfp-nuoF* (A), BW25113 *mcherry-sdhC* (B), BW25113 *cydB-egfp* (C), BW25113 *cyoA-mCherry* (D), and BW25113 *atpB-egfp* (E). White bars 1 *μ*m.

**Figure 6:**
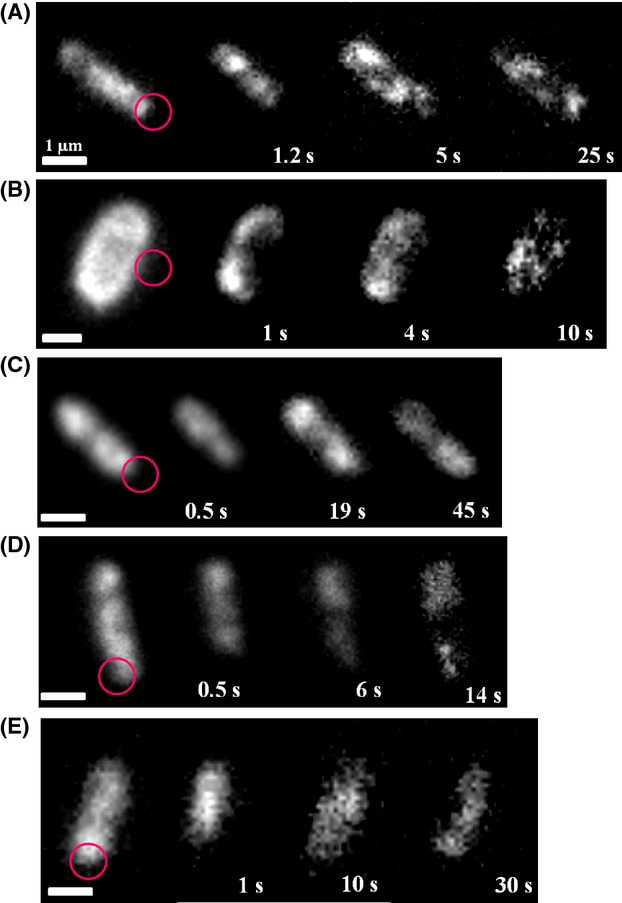
FRAP experiments using TIRF microscopy, images of *E*. *coli* strains BW25113 *egfp-nuoF* (A), BW25113 *mcherry-sdhC* (B); BW25113 *cydB-egfp* (C), BW25113 *cyoA-mCherry* (D), BW25113 *atpB-egfp* (E). The circle indicates the area of bleaching, numbers indicate time after bleaching. White bars 1 *μ*m.

The mobility of the FP-labeled OXPHOS complexes was investigated by TIRF microscopy. The images (Fig. 5) and streams showed a very similar patchy distribution pattern of the enzyme complexes to that observed by epifluorescence microscopy. The patches had the same diameter between 300 and 500 nm as determined in epifluorescence microscopy. The stream recordings revealed that the patches of the OXPHOS complexes are not static but highly dynamic (Movies S1–S4). The movement of the patches did not follow an obvious pattern and the OXPHOS complexes diffused randomly. From time to time, little droplets constricted from one patch and fused with another patch (Movies S1–S4). The dynamics of the OXPHOS complexes were investigated using FRAP experiments combined with TIRF microscopy (Fig. 6). There is a fast recovery of the fluorescence after bleaching a defined membrane area by the FRAP laser in all strains (Movies S5–S8). On average, the fluorescence recovery into the bleached area was complete after ∼10 sec. In some experiments, the bleached parts even contained the patches with the highest fluorescence emission after recovery. In most cases, the fluorescence reappeared in the bleached areas by a diffusion of an entire patch from an unbleached area. With the experimental setup, it was not possible to examine differences in the diffusion rates of the patches of fluorescence in the various strains. The observed dynamic behavior of the complexes is consistent with previous data reported for the cytochrome *bd*-I complex (Lenn et al. 2008).

## Discussion

In this study, the enzyme complexes of aerobic OXPHOS were localized in the cytoplasmic membrane of living *E. coli* cells. This was achieved by fusing the genes of either eGFP, mCerulean, or mCherry, to structural genes of respiratory complex I, complex II, cytochrome *bd*-I oxidase, cytochrome *bo* oxidase, and F_o_F_1_ ATP-synthase in the corresponding chromosomal operon. Thus, the genes were expressed and the FP-labeled proteins produced at a physiological level. This implies that the OXPHOS complexes were present in differing amounts in the membrane impeding a quantitative analysis. Considering the different fluorescence lifetime and the bleaching stability of the individual fluorophores, it was estimated that, for example, complex II is present in a more than 100-fold higher concentration in the membrane than complex I by comparison of the total fluorescence estimated for the same FP-fusion. This is most likely due to the fact that complex II is not only part of the respiratory chains but also plays a distinct role in the citric acid cycle. Due to its much higher abundance, complex II shows a more homogeneous distribution within the membrane than the other OXPHOS complexes. Accordingly, OXPHOS complexes that were present in a higher amount were observed at longer timescales than minority enzyme complexes. We were not able to determine the cellular localization of the alternative NADH dehydrogenase encoded by *ndh*, because fusions with any of the three fluorophores led to an increased fluorescence in the cytoplasm (data not shown). This is most likely due to the fact that the alternative NADH dehydrogenase is a peripheral membrane protein that forms an amphiphilic membrane-anchor by dimerization (Feng et al. 2012; Iwata et al. 2012). Attachment of a fluorophore enlarges the hydrophilic part so that the dimer might be pulled away from the membrane.

The fusions had no effect on the growth rate and led to a negligible decrease in enzymatic activity of complex I, complex II, cytochrome *bd*-I oxidase, cytochrome *bo_3_* oxidase, and F_o_F_1_ ATP-synthase. In addition, the OXPHOS complexes were fully assembled and contained the desired FP fusions. Thus, we established a system to investigate the enzyme complexes of aerobic OXPHOS in living *E. coli* cells using fluorescence microscopy techniques.

Our studies show that the aerobic OXPHOS complexes are found in large patches within the *E. coli* cytoplasmic membrane. These patches of enzyme complexes of the same kind diffused within the membrane and fluorescence moved from one patch to another indicating the dynamic composition of these patches. There was no obvious difference detectable in the mobility of the individual enzyme complexes. Similar findings have been reported for the *E. coli* cytochrome *bd*-I oxidase (Lenn et al. 2008) and the *Bacillus subtilis* ATP-synthase and succinate:ubiquinone oxidoreductase (Johnson et al. 2004; Meredith et al. 2008). Therefore, the organization of the OXPHOS complexes in larger patches seems to be a general feature at least in gram-negative *E. coli* and gram-positive *B. subtilis* (see, however, discussion below). In all FRAP experiments, the bleached area recovered fluorescence much faster than possible due to de novo protein synthesis (Ogle and Ramakrishnan 2005). Thus, the FRAP is due to the diffusion of the enzyme complexes in the membrane. The difference in the molecular mass of the OXPHOS complexes should be reflected in different diffusion coefficients. However, the rates of fluorescence recovery were virtually identical in all strains, making it very likely that the OXPHOS complexes form larger supramolecular assemblies reflected in the fluorescence patches that are obscuring the determination of individual diffusion coefficients. The same patchy distribution of the OXPHOS complexes was also detected in mitochondria (Muster et al. 2010). It is proposed that the fusion and fission dynamics of mitochondria favor a dynamic reassortment of the individual OXPHOS complexes (Muster et al. 2010). The same holds true for bacteria where a static number of the OXPHOS complexes per cell would hamper cell division.

The fluorescent patches in the *E. coli* cytoplasmic membrane are much larger than the respiratory supercomplexes characterized so far (Stroh et al. 2004; Boekema and Braun 2007; Dudkina et al. 2011; Davies et al. 2012). Thus, they cannot represent individual supercomplexes and to our knowledge a clustering of supercomplexes has not been reported. Using in vitro experiments, it was reported that supercomplexes are also formed in *E. coli*. The homotrimeric organization of the succinate:ubiquinone oxidoreductase detected by other groups (Yankovskaya et al. 2003; Sousa et al. 2011) fits nicely with our data obtained by sucrose gradient centrifugation. In addition, defined associations between the two NADH dehydrogenases, between the two terminal oxidases and the formate dehydrogenase as well as between cytochrome *bd*-I and succinate dehydrogenase have been proposed (Sousa et al. 2012; Sousa et al. 2013a,b). With the strains described here, it is not possible to decide whether these supercomplexes are formed in vivo.

The question is still open whether the assemblies clustered in patches consist of only one kind of enzyme complex, that might be called “segrazones” or whether they contain various OXPHOS complexes as proposed for the “respirazones” (Lenn et al. 2008). To answer this question, we are currently in the process of generating new *E. coli* strains that contain pairs of FP-labeled complexes with the FP fusions of different colors each at the same position of the enzyme complexes described here. A quantitative analysis of these strains will elucidate whether the fluorescent patches contain only one kind or several kinds of the OXPHOS enzyme complexes. However, as the cytochrome *bc*_*1*_ complex, the respiratory complex III, is the central and connecting part of most supercomplexes described so far and because this complex is missing in *E. coli,* we propose that the OXPHOS complexes in the *E. coli* membrane are indeed organized in “segrazones” and, thus, represent a novel type of supramolecular organization. This would also question the interpretation that the patches of ATP-synthase and succinate:ubiquinone oxidoreductase observed in *B. subtilis* (Johnson et al. 2004; Meredith et al. 2008) do not reflect a supramolecular structure, because this organism contains a cytochrome *bc* complex (Yu et al. 1995). Indeed, supercomplex assemblies consisting of *bc* complex, *caa*_*3*_ oxidase and most likely ATP-synthase and of succinate:ubiquinone oxidoreductase and cytochrome *aa*_3_ menaquinol oxidase (Garcia Montes de Oca et al. 2012) as well as of the *bc* and *caa*_*3*_ complexes in various stoichiometries (Sousa et al. 2013a,b) were reported to be present in *B. subtilis*. However, supercomplexes lacking *bc* complex such as a cytochrome *bd* oxidase/cytochrome *bo*_3_ oxidase/formate dehydrogenase supercomplex and a cytochrome *bd*/succinate dehydrogenase supercomplex have also been described (Sousa et al. 2012, 2013a,b). Noteworthy, all other bacterial supercomplexes reported in the literature so far contain a member of the cytochrome *bc* family (Magalon et al. 2012).

## Supporting Information

**Movies S1-S4.** The movies show streams of live cells from strains BW25113 *egfp-nuoF* (Movie S1), BW25113 *mcherry-sdhC* (Movie S2), BW25113 *cyoA-mcherry* (Movie S3) and BW25113 *atpB-egfp* (Movie S4) obtained by TIRF-microscopy. The streams show the localization of the OXPHOS complexes in clusters that are dynamic over time (Movies S1-S4).

**Movies S5-S8.** The movies show streams of live cells from strains BW25113 *egfp-nuoF* (Movie S5), BW25113 *mcherry-sdhC* (Movie S6), BW25113 *cyoA-mcherry* (Movie S7) and BW25113 *atpB-egfp* (Movie S8) obtained by TIRF microscopy in a FRAP experiment. A part of the cell was bleached with a short laser pulse and the recovery of fluorescence in the bleached areas is seen (Movies S5-S8). All OXPHOS complexes rapidly diffuse into the bleached areas with similar kinetics.
