# Supplementary material for: Organization of the Escherichia coli aerobic enzyme complexes of oxidative phosphorylation in dynamic domains within the cytoplasmic membrane
Source: Microbiologyopen. 2014 Apr 12;3(3):316–26. doi: 10.1002/mbo3.163 (PMC4082705; doi:10.1002/mbo3.163)
Supplement: Supplementary file 9 — Movies S5-S8. The movies show streams of live cells from strains BW25113 egfp-nuoF (Movie S5), BW25113 mcherry-sdhC (Movie S6), BW25113 cyoA-mcherry (Movie S7) and BW25113 atpB-egfp (Movie S8) obtained by TIRF microscopy in a FRAP experiment. A part of the cell was bleached with a short laser pulse and the recovery of fluorescence in the bleached areas is seen (Movies S5-S8). All OXPHOS complexes rapidly diffuse into the bleached areas with similar kinetics. [file mbo30003-0316-sd9.doc]

**Supplementary Information for:**

**Organization of the *E. coli* aerobic enzyme complexes of oxidative phosphorylation in dynamic domains within the cytoplasmic membrane**

**Heiko Erhardt^a^, Felix Dempwolff^b,c^, Moritz Pfreundschuh^a,d^, Marc Riehle^a,e^, Caspar Schäfer^a,f^, Thomas Pohl^a,g^, Peter Graumann^b,c^ and Thorsten Friedrich^a,1^**

**Author affiliation:** ^a^Albert-Ludwigs-Universität, Albertstraße 21, Institut für Biochemie, 79104 Freiburg, Germany, ^b^Albert-Ludwigs-Universität, Institut für Biologie II, Mikrobiologie, Schänzlestraße 1, 79104 Freiburg, Germany, ^c^Phillips-Universität, LOEWE Center for Synthetic Microbiology (SYNMICRO), Hans-Meerwein-Straße 6, 35043 Marburg, Germany, ^d^ETH Zürich, Department of Biosystems Science and Engineering, Mattenstraße 26, 4058 Basel, Switzerland, ^e^Eberhard-Karls-Universität, Institut für Pharmakologie und Toxikologie, Wilhelmstraße 56, 72074 Tübingen, Germany, ^f^Humboldt Universität zu Berlin, Institut für Biologie und Mikrobiologie, Chausseestraße 117, 10115 Berlin, Germany, ^g^SuppreMol GmbH, Am Klopferspitz 19, 82152 Martinsried, Germany.

**Corresponding author:** Thorsten Friedrich, Albert-Ludwigs-Universität, Institut für Biochemie, Albertstraße 21, 79104 Freiburg, Germany, E-mail: thorsten.friedrich@uni-freiburg.de. Tel: +49-(0)761-203-6060, Fax: +49-(0)761-203-6096.

**Materials and Methods**

*Construction of E. coli strains.* The *E. coli* strains, plasmids and oligonucleotides used in this study are listed in Tables S1, S2 and S3.

*Construction of strain BW25113 nuoF-mcerulean.* FP-fusions were generated in the chromosomal *nuo*-operon by use of subclones. Linear DNA fragments for λ–red mediated recombination were amplified from the subclones. For the C-terminal fusion of subunit NuoF with the blue fluorescent protein mCerulean (1) we used pCA24N*nuoF* (2) as host vector. The insertion fragment was amplified from pAS1Nb-*mcerulean* (1) with the oligonucleotid pair P1 and P2 and cut with *Not*I prior to ligation. For the fusion of the mCerulean coding gene to the 3’ of *nuoF*, pCA24N*nuoF* was cut with *Not*I and ligated with the insertion fragment (Fermentas). The ligated plasmid pCA24N*nuoF-mcerulean* was introduced into DH5α-cells (Invitrogen) and the success of the ligation was checked by sequencing (GATC, data not shown). From the subclone the gene of the fusion protein NuoF-mCerulean was amplified using the oligonucleotide pair P3 and P4. The linear DNA fragment was used for λ–red mediated recombination in *E. coli* strain DH5α ∆*nuo*/pBAD*nuo* *nuoF*::*nptIsacRB* (3, 4) to introduce the modified gene in episomal *nuo*-operon. The complex I expression plasmid pBAD*nuo*/*nuoF-mcerulean* was used to demonstrate the full assembly and activity of the modified complex I (data not shown). To integrate the modified *nuo*-gene in the chromosomal *nuo*-operon the appropriate DNA-sequence from pBAD*nuo*/*nuoF-mcerulean* was amplified with the oligonucleotide pair P5 and P6 to generate long homologous regions. The strain BW25113 *nuoF::nptIsacRB* was generated accordingly to the episomal insertion mutant *via* λ–red mediated recombination as described (4). The genomic identity of *E. coli* strain BW25113 *nuoF-mcerulean* was checked by colony PCR followed by sequencing of the particular DNA-fragment (GATC).

*Construction of strains BW25113 mcherry-nuoF and BW25113 egfp-nuoF.* *E. coli* strains BW25113 *mcherry-nuoF* and BW25113 *egfp-nuoF* were generated in accordance with the procedure described above. Prior to ligation a point mutation was inserted into pCA24N*nuoF* (2) *via* the oligonucleotide pair P7 and P8 eliminating a restriction site for *BamH*I. This was necessary to give access to the 5’ of *nuoF* *via* single cutting the modified plasmid pCA24N*nuoF** with *BamH*I. Oligonucleotide pair P9 and P10 was used to amplify the mCherry-coding genes from pRSET‑*mcherry* (5), oligonucleotide pair P11 and P12 was used to amplify the eGFP-coding genes from pKF2-6 (20), respectively. After restriction of the PCR-product with *BamH*I the linear DNA was ligated in pCA24N*nuoF** and used for transformation of DH5α. The success was checked by DNA-sequencing (data not shown). Linear DNA-fragments were amplified from the plasmids pCA24N*egfp-nuoF* and pCA24N*mcherry-nuoF* with the oligonucleotide pair P13 and P14 or P14 and P15, respectively. The complex I overexpression plasmid pBAD*nuo* was complemented by these fragments *via* λ–red mediated recombination in *E. coli* strain DH5α ∆*nuo*/pBAD*nuo* *nuoF*::*nptI-sacRB* (3, 4). The fluorescent complex I variants were produced from pBAD*nuo*/*egfp-nuoF* and pBAD*nuo*/*mcherry-nuoF* to checked the full assembly and activity of the variants (data not shown). The DNA-fragments for λ‑red mediated recombination in BW25113 *nuoF::nptIsacRB* were amplified by PCR with the oligonucleotide pair P16 and P17 for both mutants. The success of the recombination was checked by colony-PCR and DNA sequencing (GATC).

*Construction of strain BW25113 mcherry-sdhC.* Plasmid pSDH15 (6) contains the genes encoding the *E. coli* succinate dehydrogenase. An additional restriction site for *Bgl*II was introduced *via* PCR to the 5’ of *sdhC* with the oligonucleotide pair P22 and P23 to allow ligation with the mCherry gene, amplified with P24 and P25 from pRSET-*mcherry* (5) in the plasmid upstream of *sdhC*. The modified plasmid pSDH15* and the PCR-fragment were cut with *Bgl*II prior to ligation. DH5α cells were transformed with plasmid pSDH15*mcherry-sdhC* and checked by restriction and sequencing (GATC, data not shown). For the integration of the modified gene in the chromosomal *sdh*-operon, the *nptI-sacRB-*cartridge was amplified with the oligonucleotide pair P26 and P27 from plasmid pUM24 in BW25113 and introduced in BW25113 *via* λ–red mediated recombination (4) creating BW25113 *sdhC::nptIsacRB*. This correct genotype was checked by colony PCR (data not shown) and used for the second step of recombination in which the full *sdh*-operon was established with the modified gene amplified with the oligonucleotide pair P28 and P29 from pSDH15*mcherry‑sdhC*. Strain BW25113 *mcherry-sdhC* was checked by colony-PCR and DNA sequencing (GATC, data not shown).

*Construction of strain BW25113 cydB-egfp (YTL01).* *E. coli* strain YTL01 was a generous gift from Drs. T. Lenn and C. Mullineaux (Queen Mary University of London). The construction of this strain is described in (7). YTL01 contains an eGFP-Fusion on *cydB* in *E. coli* BW25113.

*Construction of strain BW25113 cyoA-mcherry.* The chromosomal *cyo*-operon was inactivated by insertion the *nptI-sacRB-*cartridge amplified with the oligonucleotide pair P30 and P31 from plasmid pUM24 in BW25113 *via* λ–red mediated recombination (4). The correctness of strain BW25113 *cyoA::nptIsacRB* was checked by colony PCR (data not shown). The *cyo*-operon of this strain was re-established in a second recombination step using the modified gene amplified with the oligonucleotide pair P32 and P33 from pRSET-*mcherry* (5). Strain BW25113 *cyoA-mCherry* was checked by colony-PCR followed by sequencing (GATC, data not shown).

*Construction of strain BW25113 atpB-egfp.* Plasmid pKF2-6 contains all genes for the F_o_F_1_ ATP-synthase including a C-terminal fusion of eGFP to AtpA (*atpB;* 20). The *nptI-sacRB* cartridge was inserted in the chromosomal *atp*-operon replacing *atpB* *via* λ–red mediated recombination (4). The cartridge was amplified from plasmid pUM24 using the oligonucleotide pair P18 and P19 imparting homologous regions to the chromosomal *atp*-operon. Strain BW25113 *atpB::nptI-sacRB* was checked by colony PCR (data not shown). For the insertion of the gene coding the fluorescent labeled subunit AtpA a linear fragment was amplified from pKF2-6 using the oligonucleotide pair P20 and P21. The fragment was integrated into the chromosome of BW25113 *atpB::nptI-sacRB* *via* λ‑red mediated recombination (4). Strain BW25113 *atpB-egfp* was checked by colony PCR and DNA sequencing (GATC, data not shown).

*Cell growth.* Cells that were used for the preparation of cytoplasmic membranes were grown as 400 ml LB broth in 1L baffled conical flasks at 30°C. The cells were harvested when entering the stationary phase by centrifugation at 4’480 g, 4°C and stored at -80°C until use. For fluorescence microscopy 4 ml S7_50_ cultures were inoculated in a 1:100 ratio (v:v) with a 4 ml LB overnight culture and grown at 30°C. If necessary, the media were supplemented with 100 µg/ml ampicillin, 170 µg/ml (liquid media) or 20 µg/ml (agar-plates) chloramphenicol and 50 µg/ml kanamycin.

*Preparation of cytoplasmic membranes.* For the preparation of the cytoplasmic membranes 6-10 g cells (wet mass) were suspended in 20 ml 50 mM MES/NaOH, 50 mM NaCl, pH 6.0 with 10 μg/ml DNase I (Boehringer), 0.1 mM phenylmethanesulfonyl fluoride (Sigma) and disrupted by a single pass through a French pressure cell (SLM Aminco) at 110 MPa. Cell debris was removed by centrifugation for 20 min at 36’000 g and the supernatant was centrifuged for 1 h at 250 000 g. The sediment was treated as fraction of cytoplasmic membranes. The membranes were suspended in 50 mM MES/NaOH, 50 mM NaCl, pH 6.0 at a protein concentration of 60-80 mg/ml.

*Enzyme activities.* The NADH oxidase activity of cytoplasmic membranes was measured with a Clarke-type oxygen electrode at 30°C in a volume of 2 ml 50 mM Mes/NaOH, 50 mM NaCl, 5 mM MgCl2, pH 6.0. The assay contained 2-3 mg cytoplasmic membranes and the reaction was started by an addition of 1.25 mM NADH (8). The succinate oxidase activity was measured with the same experimental setup. After an incubation of 30 min at 30°C, the reaction was started by an addition of 10 mM succinat (9).

The NADH/ferricyanide oxidoreductase activity was measured in 1 ml 50 mM MES/NaOH, 50 mM NaCl, 1 mM K3[Fe(CN)6], 0.2 mM NADH, pH 6.0 as described (10). The succinate/ferricyanide oxidoreductase activities were measured at 30°C in 1 ml 50 mM phosphate buffer, pH 7.0 containing 0.45 mM K3[Fe(CN)6] and 10 mM succinate (11). The reactions were started by the addition of 10 µl membrane suspensions or 30 µl of a sucrose gradient fraction.

The activity of the modified F_o_F_1_ ATP-synthase was checked by the ability of strain BW25113 *atpB-egfp* to grow on minimal media plates containing succinate as sole carbon source (12).

*Sucrose gradient centrifugation.* Membrane proteins were solubilized from the cytoplasmic membranes by an addition of 3 % (w/v) *n*‑dodecyl-β-D-maltopyranoside (DDM, AppliChem) at 4°C. The suspension was incubated for 10 min at 4°C and gently homogenized from time to time. The extract was centrifuged for 20 min at 48’000 g and 4°C to remove non-solubilized material. 0.9 ml aliquots of the supernatant were loaded onto 12 ml gradients of 5-30 % (w/v) sucrose in 50 mM MES/NaOH, 50 mM NaCl, and 0.1 % (w/v) DDM, pH 6.0 and centrifuged for 18 h at 160’000 g. The gradients were fractionated into 0.7 ml portions and the NADH/ferricyanide oxidoreductase activity (strains with a modified *nuo*-operon) and the succinate/ferricyanide oxidoreductase activity (BW25113 *mcherry-sdhC*) of the fractions were measured. In addition, the fluorescence emission of the fractions was determined using the specific wavelengths of the particular fluorescent proteins (mCerulean: λ_EX_ 430 nm; λ_EM_ 475 nm, eGFP: λ_EX_ 480 nm; λ_EM_ 510 nm, mCherry: λ_EX_ 587 nm; λ_EM_ 610nm). 100 µl of each fraction were diluted with 900 µL 30 mM NaH_2_PO_4_, pH 7.4 and the fluorescence was measured in 1 ml cuvette (Hellma, 10 mm) with a fluorescence spectrometer (SFM 25, Kontron). The activity and the fluorescence determined for each fraction were normalized according to the amount of protein applied on the gradient. The data are shown in Figure S2.

**Table S1.** Oligonucleotides.

| Oligonucleotide | Sequence |
| --- | --- |
| P1 | 5’-TATGCGGCCGCAGCAAGGGCGAGGAGCTGTTCAC-3’ |
| P2 | 5’-TTAGCGGCCGCTTACTTGTACAGCTCGT CATGCCGAG-3’ |
| P3 | 5’-AACATTATCCGTACTCCCGAAACG-3’ |
| P4 | 5’-GCTTCCAGTTTTCTCAGTCAGAGACTGAGCGTTAATCGAAATTCGGTTACTTGT ACAGCTCGTCCATGC-3’ |
| P5 | 5’-GGTGTGGCAACGTTCTACAG-3’ |
| P6 | 5’-CTTCACATACCGGACAGTCG-3’ |
| P7 | 5’-GATAATAATGGTTTCTTAGGGCCCTGATGTCCGGCGGTGCTTTTG-3’ |
| P8 | 5’-CAAAAGCACCGCCGGACATCAGGGCCCTAAGAAACCATTATTATC-3’ |
| P9 | 5’-TAGGATCCGAGCAAGGGCGAGGAGGATAACATG-3’ |
| P10 | 5’-ATGGATCCGTCTTGTACAGCTCGTCCATGCCG-3’ |
| P11 | 5’-TAGGATCCGAGTAAAGGAGAAGAACTTTTCACTGGA-3’ |
| P12 | 5’-ATGGATCCGTTTTGTATAGTTCATCCATGCCATGTGTAATC-3’ |
| P13 | 5’-TCTGACCCCGGAAGCGATCCCTGAACTGCTGGAGCGGTATAAATGAGCAAGGG CGAGGAGGATAACATG-3’ |
| P14 | 5’-CAGATCAAGGTGCGCTTC-3’ |
| P15 | 5’-CTGACCCCGGAAGCGATCCCTGAACTGCTGGAGCGGTATAAATGAGTAAAGGA GAAGAACTTTTCACTG-3’ |
| P16 | 5’-GGTGTGGCAACGTTCTACAG-3’ |
| P17 | 5’-CTTCACATACCGGACAGTCG-3’ |
| P18 | 5’-GGTCTGTTGCCGTGGTGGTCACAGTGGATCCTGAATGTGCCGTCA-3’ |
| P19 | 5’-GTTTTCCATGACAGTCTCCAGTTTGTTTCAGTTAAAACGTAGTAG-3’ |
| P20 | 5’-TGGCACTTGGCGTATTTATCC-3’ |
| P21 | 5’-TACTACGCGACAGCGAACATC-3’ |
| P22 | 5’-GTGGGCGTTATTCATGATAAGATCTGTGAAAAAACAAAGACCTGTTAATCTGG-3’ |
| P23 | 5’-CCAGATTAACAGGTCTTTGTTTTTTCACAGATCTTATCATGAATAACGCCCAC-3’ |
| P24 | 5’-ACGAGATCTGTGAGCAAGGGCGAGGAGG-3’ |
| P25 | 5’-TTAAGATCTCAGGGCCGGATCCGTCTTGTACAGCTCGTCCATGCC-3’ |
| P26 | 5’-GTAGTCCCCAGGGAATAATAAGAACAGCATGTGGGCGTTATTCCACGTTGTGT CTCAAAATCTCTGATG-3’ |
| P27 | 5’-GGTACCCAGAAGCCACAGCAGGATGCCCACTGCAACAAAGGTGATCACACGA ATTCCCCGGGGGATCCG-3’ |
| P28 | 5’-GCCACCCAGCGTTGTAAC-3’ |
| P29 | 5’-GCGTTGCTTACCATACGAGG-3’ |
| P30 | 5’‑CACGAAGGTATGGAAGGCATGGACATGAGCCACGCGGAATCCGCCCATCACGTTGTGTCTCAAAATCTCTGATG-3’ |
| P31 | 5’‑CTGCATCAAGTGATAATTTTCCGAACATCTTTATTCTTCCTCAACCCCTGGAATTCCCCGGGGGATCCG-3’ |
| P32 | 5’‑CACGAAGGTATGGAAGGCATGGACATGAGCCACGCGGAATCCGCCCATATGGTGAGCAAGGGCGAGGAG-3’ |
| P33 | 5’‑CTGCATCAAGTGATAATTTTCCGAACATCTTTATTCTTCCTCAACCCCTCTACTTGTACAGCTCGTCCATG--3’ |

**Table S2.** Plasmids.

| Plasmid | Genotype | Reference |
| --- | --- | --- |
| pCA24N*nuoF* | cam^R^, ColE1 origin, *lacI-*P_T5-_*_lac_* His_6_-*nuoF* | (2) |
| pCA24N*nuoF** | cam^R^, ColE1 origin, *lacI-*P_T5-_*_lac_* His_6_-*nuoF BamH*I(2009)::*Apa*I | This work |
| pAS1Nb-*mcerulean* | amp^R^, pUC origin, *mcerulean* | (5) |
| pRSETb-*mcherry* | amp^R^, pRSETb, *mcherry* | (5) |
| pkF2-6 | amp^R^, *atpA-I*´, *atpA-gfp* | (20) |
| pKD46 | amp^R^, R101 origin, *araC*-P*_araBAD_ exo, bet, gam* | (19) |
| pUM24 | amp^R^, kan^R^, *nptI-sacRB* | (4) |
| pBAD*nuo* *nuoF*::*nptIsacRB* | cam^R^, p15a origin, *araC-*P*_araBAD_ nuoA-N*, *nuoF::nptI-sacB* | (4) |
| pBAD*nuo*/*nuoF-mCerulean* | cam^R^, p15a origin, *araC-*P*_araBAD_ nuoA-N*, *nuoF-mcerulean* | This work |
| pBAD*nuo*/*mcherry-nuoF* | cam^R^, p15a origin, *araC-*P*_araBAD_ nuoA-N*, *mcherry-nuoF* | This work |
| pBAD*nuo*/*egfp-nuoF* | cam^R^, p15a origin, *araC-*P*_araBAD_ nuoA-N*, *egfp-nuoF* | This work |
| pSDH15 | amp^R^, pBR322, *sdhC*^+^*D*^+^*A*^+^*B*^+^ | (6) |
| pSDH15* | amp^R^, pBR322, *sdhC*^+^*D*^+^*A*^+^*B*^+^ , +*Bgl*II(935) | This work |
| pSDH15*mcherry-sdhC* | amp^R^, pBR322, *sdhC*^+^*D*^+^*A*^+^*B*^+^, *mcherry-sdhC* | This work |

**Table S3.** Strains used in this study.

| *E. coli* strain | Genotype | Reference |
| --- | --- | --- |
| DH5α | *sup*E44 Δ*lac*U169 (Φ80*lac*ZΔM15)  *hsd*R17 (rk- mk+) *rec*A1 *end*A1  *gyr*A96 *thi-1 rel*A1 | Invitrogen |
| DH5α ∆*nuo* | DH5α ∆*nuo* | (21) |
| BW25513 | lacIq rrnB3 ΔlacZ4787 hsdR514  Δ(araBAD)567 Δ(rhaBAD)568 rph-1 | (19) |
| BW25113 *nuoF::nptIsacRB* | BW25113 *nuoF::nptIsacRB* | (4) |
| BW25113 *atpE::nptIsacRB* | BW25113 *atpE::nptI-sacRB* | This work |
| BW25113 *sdhC::nptIsacRB* | BW25113 *sdhC::nptIsacRB* | This work |
| BW25113 *nuoF-mcerulean* | BW25113 *nuoF-mcerulean* | This work |
| BW25113 *mcherry-nuoF* | BW25113 *mcherry-nuoF* | This work |
| BW25113 *egfp-nuoF* | BW25113 *EGFP-nuoF* | This work |
| BW25113 *atpB-egfp* | BW25113 *atpB-egfp* | This work |
| BW25113 *mcherry-sdhC* | BW25113 *mcherry-sdhC* | This work |
| BW25113 *cyoA-mcherry* | BW25113 *cyoA-mcherry* | This work |
| BW25113 *cydB-egfp* (YTL01) | BW25113 *cydB-egfp* | (7) |

**
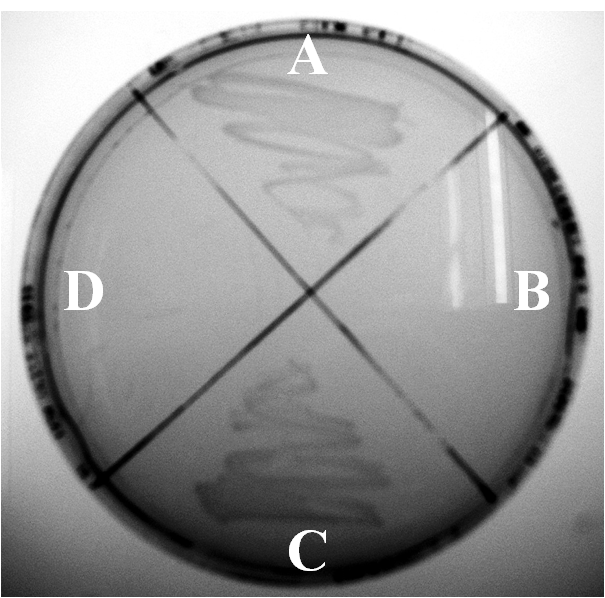
**

**Fig. S1.** Growth of various *E. coli* strains containing mutations in the *atp*-operon on agar plates with M9 minimal medium plus succinate as sole carbon source. A) BW25113; C) BW25113 *atpB-egfp* ; B) and D) BW25113 *atpB::nptIsacRB*.

**References:**

1. Rizzo, M.A., Springer, G.H., Granada, B., and Piston, D.W. (2004) An improved cyan fluorescent protein variant useful for FRET. *Nat Biotechnol* **22**: 445–449.

2. Kitagawa, M. et al. (2005) Complete set of orf clones of *Escherichia* *coli* aska library (a complete set of e. coli k-12 orf archive): unique resources for biological research. *DNA* *Res* 12:291–299.

3. Spehr, V., Schlitt, A., Scheide, D., Guénebaut, V., and Friedrich, T. (1999) Overexpression of the *Escherichia* *coli* *nuo*-operon and isolation of the overproduced NADH:ubiquinone oxidoreductase (complex I). *Biochemistry* **38**: 16261–16267.

4. Pohl, T., Uhlmann, M., Kaufenstein, M., and Friedrich, T. (2007) Lambda red-mediated mutagenesis and efficient large scale affinity purification of the *Escherichia* *coli* NADH:ubiquinone oxidoreductase (complex I). *Biochemistry* **46**: 10694–10702.

5. Shaner, N.C. *et al*. (2004) Improved monomeric red, orange and yellow fluorescent proteins derived from *Discosoma* *sp*. red fluorescent protein. *Nat* *Biotechnol* **22**: 1567–1572.

6. Vibat, C.R., Cecchini, G., Nakamura, K., Kita, K.,and Gennis, R.B. (1998) Localization of histidine residues responsible for heme axial ligation in cytochrome *b*_556_ of complex ii (succinate:ubiquinone oxidoreductase) in *Escherichia* *coli*. *Biochemistry* **37**: 4148–4159.

7. Lenn, T., Leake, M.C., and Mullineaux, C.W. (2008) Clustering and dynamics of cytochrome *bd*-I complexes in the *Escherichia* *coli* plasma membrane *in* *vivo*. *Mol* *Microbiol* **70**: 1397–1407.

8. Flemming, D., Hellwig, .P, and Friedrich, T. (2003) Involvement of tyrosines 114 and 139 of subunit NuoB in the proton pathway around cluster N2 in *Escherichia* *coli* NADH:ubiquinone oxidoreductase. *J Biol Chem* **278**: 3055–3062.

9. Pohl, T. *et al*. (2007a) Effects of the deletion of the *Escherichia* *coli* frataxin homologue CyaY on the respiratory nadh:ubiquinone oxidoreductase. *BMC* *Biochem* **8**: 13.

10. Friedrich, T. *et al*. (1989) A small isoform of NADH:ubiquinone oxidoreductase (complex I) without mitochondrially encoded subunits is made in chloramphenicol-treated *Neurospora* *crassa*. *Eur* *J* *Biochem* **180**: 173–180.

11. Maklashina, E., and Cecchini, G. (1999) Comparison of catalytic activity and inhibitors of quinone reactions of succinate dehydrogenase (succinate-ubiquinone oxidoreductase) and fumarate reductase (menaquinol-fumarate oxidoreductase) from *Escherichia* *coli*. *Arch* *Biochem* *Biophys* **369**: 223–232.

12. Johnson, A.S., van Horck, S., and Lewis, P.J. (2004) Dynamic localization of membrane proteins in *Bacillus* *subtilis*. *Microbiology* **150**: 2815–2824.

13. Leif, H., Sled, V.D., Ohnishi, T., Weiss, H., and Friedrich, T. (1995) Isolation and characterization of the proton-translocating nadh: ubiquinone oxidoreductase from *Escherichia* *coli*. *Eur* *J* *Biochem* **230**: 538–548.

14. Melo, A.M.P., Bandeiras, T.M., and Teixeira, M. (2004) New insights into type ii NAD(P)H:quinone oxidoreductases. *Microbiol Mol Biol Rev* **68**: 603–616.

15. Braun, M., Bungert, S., and Friedrich, T. (1998) Characterization of the overproduced NADH dehydrogenase fragment of the NADH:ubiquinone oxidoreductase (complex I) from *Escherichia* *coli*. *Biochemistry* 37:1861–1867.

16. Yankovskaya, V. *et al.* (2003) Architecture of succinate dehydrogenase and reactive oxygen species generation. *Science* **299**: 700–704.

17. Laemmli, U.K. (1970) Cleavage of structural proteins during the assembly of the head of bacteriophage t4. *Nature* **227**: 680–685.

18. Blas, A.L.D., and Cherwinski, H.M. (1983) Detection of antigens on nitrocellulose paper immunoblots with monoclonal antibodies. *Anal Biochem* **133**: 214–219.

19. Datsenko, K.A., and Wanner, B.L. (2000) One-step inactivation of chromosomal genes in *Escherichia* *coli* K-12 using PCR products. *Proc Natl Acad Sci U S A* **97**: 6640–6645.

20. Düser, M.G., Bi, Y., Zarrabi, N., Dunn, S.D., and Börsch, M. (2008) The Proton-translocating *a* Subunit of F_o_F_1_-ATP Synthase Is Allocated Asymmetrically to the Peripheral Stalk. *J. Biol. Chem*. **283**: 33602-33610.

21. Pohl, T., Bauer, T., Dörner, K., Stolpe, S., Sell, P., Zocher, G., and Friedrich, T. (2007) Iron-sulfur cluster N7 of the NADH:ubiquinone oxidoreductase (complex I) is essential for stability but not involved in electron transfer. *Biochemistry* **46**: 6588–6596.
