# Supplementary material for: Organization of the Escherichia coli aerobic enzyme complexes of oxidative phosphorylation in dynamic domains within the cytoplasmic membrane
Source: Microbiologyopen. 2014 Apr 12;3(3):316–26. doi: 10.1002/mbo3.163 (PMC4082705; doi:10.1002/mbo3.163)
Supplement: Supplementary file 10 — Movies S5-S8. The movies show streams of live cells from strains BW25113 egfp-nuoF (Movie S5), BW25113 mcherry-sdhC (Movie S6), BW25113 cyoA-mcherry (Movie S7) and BW25113 atpB-egfp (Movie S8) obtained by TIRF microscopy in a FRAP experiment. A part of the cell was bleached with a short laser pulse and the recovery of fluorescence in the bleached areas is seen (Movies S5-S8). All OXPHOS complexes rapidly diffuse into the bleached areas with similar kinetics. [file mbo30003-0316-sd10.docx]

Legend to Movies S1 to S4:

The movies show streams of live cells from strains BW25113 *egfp-nuoF* (Movie S1), BW25113 *mcherry-sdhC* (Movie S2), *BW25113* *cyoA-mcherry* (Movie S3) and BW25113 *atpB-egfp* (Movie S4) obtained by TIRF-microscopy. The streams show the localization of the OXPHOS complexes in clusters that are dynamic over time (Movies S1-S4).
